# Prevalence of self-reported work-related illness and injuries among building construction workers, Shiraz, Iran

**DOI:** 10.17179/excli2018-1459

**Published:** 2018-07-25

**Authors:** Milad Derakhshan Jazari, Mehdi Jahangiri, Hamed Khaleghi, Narges Abbasi, Soheil Hassanipour, Mahnaz Shakerian, Mojtaba Kamalinia

**Affiliations:** 1 Student Research Committee, Department of Occupational Health Engineering, School of Health, Shiraz University of Medical Science, Shiraz, Iran; 2 Research Center for Health Sciences, Institute of Health, Department of Occupational Health Engineering, School of Health, Shiraz University of Medical Sciences, Shiraz, Iran; 3 Gastrointestinal & Liver Diseases Research Center (GLDRC), Guilan University of Medical Sciences, Rasht, Iran; 4 Department of Occupational Health Engineering, School of Health, Shiraz University of Medical Sciences, Shiraz, Iran

**Keywords:** prevalence, work-related injuries, work-related illness, construction workers

## Abstract

The construction industry is one of the largest and most hazardous industries in the world, which has a direct role in the development of countries. The purpose of this study was to investigate the prevalence of self-reported work-related illness and injuries among construction workers in Shiraz, Iran. 850 randomly selected workers from 2450 construction sites completed a self-statement questionnaire regarding the prevalence of self-reported work-related illness and injuries (WRIIs), in Shiraz, Iran. The association of WRII with demographic variables were studied. The overall prevalence rate of occupational injuries was 31 %. Musculoskeletal disorders (53.3 %), eye diseases (34.1 %) and skin diseases (30.1 %) have been the most prevalent work-related illnesses among construction workers, respectively. The prevalence of WRIIs among construction workers was significantly associated with age, education, marriage, work experience, safety training programs and number of workers in the workplace as well as employment status. Considering the high prevalence of WRIIs among construction workers, more stringent occupational safety and health interventions are recommended in construction workplaces.

## Introduction

The construction industry is one of the largest and most hazardous industries in the world, which has a direct role in the development of countries. Construction workers are exposed to work-related health and safety hazards since this industry is not well organized in developing countries due to its rapid growth (Bhuiyan et al., 2016[6]; Biswas et al., 2017[7]; Murie, 2007[24]). 

There are very specific potential hazards in this industry including working at high altitude, working with power transmission equipment, continuous work change, employing contract workers instead of permanent employees, the presence of several uncoordinated contractors in a construction site and inappropriate working conditions in terms of exposure to various harmful factors such as noise, vibration, aerosols, manual handling, etc. (Carter and Smith, 2006[11]; Pinto et al., 2011[25]; Tam et al., 2001[32]).

Studies have shown that work-related injuries are significantly associated with various factors including lack of safety and health training programs, employing young workers (Aderaw et al., 2011[2]), low literacy among workers (Kunar et al., 2008[20]), smoking (Bhattacherjee et al., 2003[5]), sleep problems (Salminen et al., 2010[27]), long working hours, working at night, low work experience (Dembe et al., 2005[13]), high physical activity without exercising at all (Smith and Mustard, 2004[30]) and not using personal protective equipment (Kumar et al., 2010[19]). It is noteworthy that health consequences of these harmful factors in developing countries are 10-20 times higher than those in industrialized developed countries.

About 350 million workers currently work in this industry around the world (Biswas et al., 2017[7]). While in developed countries approximately 6-10 % of the workers are employed in the construction industry, about 20-40 % of deaths are attributed to this industry (Raheem and Hinze, 2014[26]). For example, despite the fact that 7.7 % of the workers in the United States are employed in the construction industry, 22.2 % of work-related mortalities occur in this industry (Bureau of Labor Statistics, 2004[10]). It seems that injuries among construction workers happen more frequently in developing countries compared with developed countries (Amiri et al., 2014[4]). According to the statistics presented by Hong Kong Labor department, the highest work-related fatality rate over the past decade has been related to the construction industry so that in 2015, 32.38 % of industrial accidents and 79.17 % of total work-related deaths occurred in the construction industry (Man et al., 2017[21]). Also, in Turkey, annual work-related accidents have reached the threat level and 400 deaths as well as 400 total disabilities have emerged out of 6,000-9,000 work-related accidents. Moreover, it has been reported that work-related accidents which lead to death are increasing in this industry (Yilmaz, 2014[36]). In Iran, almost 37 % of industrial accidents occur in the construction industry, while only 14 % of the workers work in this industry (Amiri et al., 2014[4]).

The construction workers are exposed not only to hazardous equipment, machinery and situations but also to work-related diseases due to workplace health problems such as harmful factors including physical factors (noise, vibration, thermal stress), chemical factors (aerosols, gases and vapors) and ergonomic factors (manual handling, improper body positioning, exerting excessive strength and repetitive movements) (Bhuiyan et al., 2016[6]; Van der Molen, 2016[33]; Wang et al., 2017[34]). However, limited studies have been conducted on the prevalence of work-related diseases among construction workers. In studies on construction workers, the prevalence of work-related diseases among construction workers in Netherlands has been about 13 % in 2010-2014 (Bock et al., 2003[8]). Various studies have reported respiratory (Bock et al., 2003[8]), ocular (Alazab, 2004[3]), skin (Bhuiyan et al., 2016[6]), and neurological diseases (Boschman et al., 2013[9]) as well as musculoskeletal disorders (Boschman et al., 2013[9]; Siu et al., 2004[29]) as the most common work-related diseases in the construction industry in other countries. In Iran, there is no information available on work-related diseases in this industry.

Given that there is no comprehensive and complete information based on actual recorded data on the prevalence of work-related illness and injuries (WRII) in the construction industry in Iran, the purpose of this study was to identify the information gap on the prevalence of WRII among construction workers in Shiraz in order to provide correction and control strategies. 

## Material and Methods

### Research design: time and space 

This cross-sectional study was conducted to investigate the prevalence of WRII among construction workers in Shiraz in 2017. Shiraz is a city in Iran with many active construction workplaces. 100 construction workplaces were selected out of 2450 workplaces in northern, southern, eastern, western and central parts of Shiraz (167,000 workers) using a Random Number Table. 

### Participants

850 workers with at least one year of work experience in the construction workplaces participated in the study including: plaster worker, ceramic tile worker, armature fixing worker, electrical worker, welder, plumber, masonry worker, laborer, painter, cement worker, and other tasks.

In cases where the workers of a workplace refused to participate in the study or quitted their work during the data collection period, the workplace was excluded from the study and was replaced by another one. 

### Data collection tools and quality control

Data was collected by researcher-made questionnaire containing questions about the prevalence of WRII, demographic characteristics of the study participants and occupational safety and health status of the workplace. The formal validity of the questions was confirmed by 30 occupational health and safety experts. For this purpose, questionnaires were sent to experts by e-mail and they gave us their feedback regarding the necessary corrections. The questionnaires were completed through interview by the first three authors. The data collection took three months. Confidentiality was maintained and informed consent was obtained. The workers were told that the collected data was just for the purpose of conducting a scientific study and they could discontinue participation in the study whenever they wished.

During training of data collectors and supervisors, issues such as the data collection instrument, field methods, inclusion-exclusion criteria and recordkeeping we emphasized. The researchers coordinated the interview process, and spot-checked and reviewed the completed questionnaires on a daily basis to ensure the completeness and consistency of the data collected. The interview questionnaire was pre-tested on 20 respondents in order to identify potential problem areas, unanticipated interpretations and cultural objections to any of the questions. Based on the pre-test results, the questionnaire was adjusted contextually.

### Data analysis 

Data analysis was performed using SPSS software version 20. Frequency distribution, mean, standard deviation and percentage were reported for each variable. The normality of each variable was then tested using Kolmogorov-Smirnov test with the error rate of ≥0.05. Chi-square and Pearson parametric tests were used to determine factors associated with WRII. The odds ratio (OR) was also presented with a 95 % confidence interval (CI) for significant variables. For multiple-comparison, Bonferroni correction was conducted by dividing the original α-value by the number of analyses on the dependent variable.

## Results

Table 1(Tab. 1) shows the demographic characteristics of the construction workers in the studied workplaces. The mean and standard deviation for age and work experience was 37.5 ± 10.7 and 10.6 ± 4.9 years, respectively. The mean and standard deviation for weekly working hours was 60±3.5 hours, and 52 % of the construction workplaces had health and safety officers.

**Figure 1:**
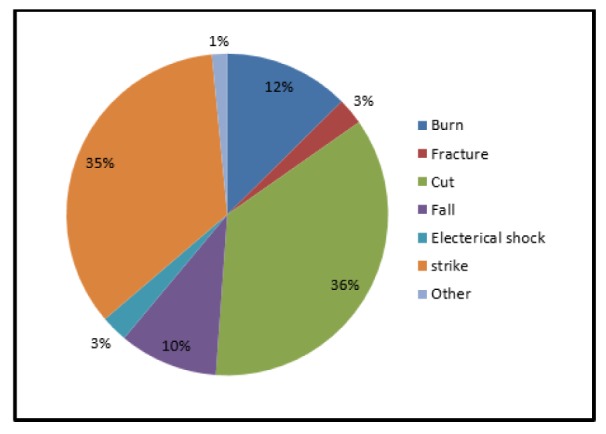
Types of self-reported work-related accidents among studied construction workers

In this study, the prevalence rate of work-related injuries was 31 %. Lacerations (36 %) and contact with heavy objects (35 %) were the most frequent causes of work-related injuries (Figure 1(Fig. 1)). 

After applying the Bonferroni test, the alpha value changed for multiple comparisons in this way from 0.05 to 0.05 / 7 = 0.007. However, our results did not change by applying Bonferroni correction because all p-values indicate highest significance (p-value < 0.001). 

Table 2(Tab. 2) presents the association between work-related illnesses and demographic characteristics of the construction workers in this study. As can be seen, WRII are significantly associated with age, work experience and number of workers in the workshop (p<0.001). There was a significant difference between the participants in our study based on age and work experience and its relationship with the prevalence of work-related injuries (p<0.001). The highest prevalence of work-related injuries was observed in people aged 21 to 40 with a prevalence of 67 % and those with a work experience of 6 to 10 years with a prevalence of 35 %.

The highest injury prevalence rate has been reported among workers aged 21-40. Also, according to the results of this study, the highest prevalence of work-related injuries has been reported among workers with a work experience of 6-10 years. As shown in Table 2(Tab. 2), most work-related injuries have occurred in workplaces of fewer than 10 workers. In this study, there was a significant association between educational level and the prevalence of WRII (p < 0.001). As the educational level increased, the prevalence of WRII decreased. The highest prevalence of WRII was reported among workers without high school diploma. As can be seen, the prevalence of all work-related diseases had a significant association with age and work experience (p < 0.001). The prevalence of work-related diseases increased with an increase in age and work experience. The highest prevalence of work-related diseases was observed among workers with a work experience of more than 11 years and those aged 21-60. The highest prevalence of musculoskeletal disorders was observed in these ranges of age and work experience. There was a significant association between the number of workplace workers and the prevalence of work-related diseases among them (p < 0.001). Therefore, the highest number of work-related illnesses was reported in workplaces with fewer than 10 workers (Table 2(Tab. 2)).

According to Table 3(Tab. 3), the prevalence of WRII had an inverse association with health and safety training programs so that workers attending safety training programs have experienced a three-fold decrease in work-related injury or illness compared with other workers [OR=0.31. CI (0.23-0.42)]. Generally, workers attending a safety training program have experienced a decreased rate of work-related illnesses. The use of PPE had a significant association with the prevalence of work-related injuries and most of the illnesses. The prevalence of injuries [OR = 0.62, 95 % CI (0.47-0.98)], musculoskeletal disorders [OR = 0.57, 95 % CI (0.36-0.74)], and psychological disorders [OR = 0.67, 95 % CI (0.46-0.97)] among the workers who used PPE was 1.5 times lower than that among other workers. 

The prevalence rate of work-related injuries among seasonal workers was 2.5 times higher than that among permanent workers [OR = 2.33; 95 % CI (1.72-3.15)]. Moreover, Seasonal workers experienced more work-related illnesses compared with permanent workers. 

In this study, the rate of work-related injuries among single workers was 5 times higher than those among married ones [OR = 0.22, 95 % CI (0.13-0.37)]. Moreover, the rate of work-related illnesses among single workers was at least twice the rate of those illnesses among married workers. Unexpectedly, in this study, a significant association was found between periodic health examinations and musculoskeletal, skin and eye diseases (p<0.05) so that people who had conducted periodic health examinations were more likely to develop musculoskeletal, skin and eye diseases.

From the workers' point of view, the most harmful factors in construction workplaces were ergonomic (85.3 %), biological (50 %), physical (46.2 %), and chemical (31 %), respectively. Among the ergonomic, physical and chemical factors, the most harmful factors were reported to be prolonged standing and improper working postures (99 %), vibration (59 %) and aerosols (63 %), respectively.

**Figure 2:**
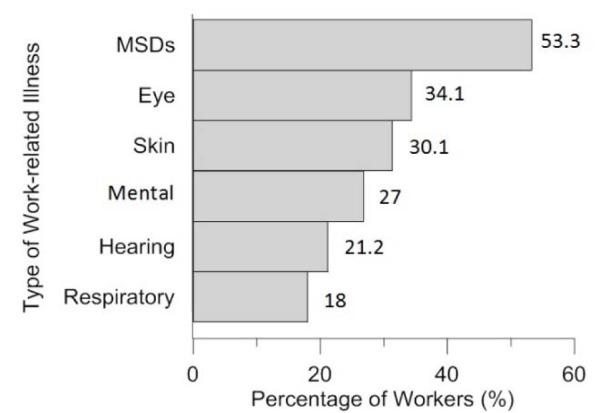
Prevalence of work-related illnesses among studied construction workers (n = 850)

Figure 2(Fig. 2) shows the prevalence of work-related illnesses among construction workers. As can be seen, musculoskeletal disorders (53.3 %) and eye diseases (34.1 %) have been the most prevalent work-related illnesses among construction workers, respectively.

## Discussion

The purpose of this study was to investigate the prevalence of self-reported WRII among construction workers in Shiraz and its association with demographic characteristics. According to the results of this study, the overall prevalence rate of work-related injuries was 31 %. Musculoskeletal disorders (53.3 %), eye diseases (34.1 %) and skin diseases (30.1 %) have been the most prevalent work-related illnesses among construction workers, respectively. The prevalence of WRII among construction workers was significantly associated with age, education, marriage, work experience, safety training programs, and number of workers in the workplace as well as employment status.

In this study, the overall prevalence rate of work-related injuries was 31 %. In various studies in other countries, the prevalence rate of work-related injuries varied from 30 % in Turkey (Gürcanli and Müngen, 2013[14]) to 38 and 84 % in Ethiopia (Adane et al., 2013[1]; Mersha et al., 2017[22]). Moradinazar et al. (2013[23]), in Ilam (west of Iran), reported the prevalence rate of work-related injuries among construction workers to be 71 %. The difference in the prevalence rate of work-related injuries can be due to factors such as differences in demographic characteristics of workers, different sample size and research method. For example, in Adane’s study, the mean age and work experience was 25.5 and 1.8 years while in the present study, the mean age and work experience was 37.5 and 10.6 years, respectively. On the other hand, in our study, the data was collected using a self-reporting method that can be affected by recall bias. Various studies have shown contradictory results regarding the repetition and diversity of work-related injuries among construction workers. For example, in some studies, slips and falls from height were the most prevalent work-related injuries (Hatami et al., 2017[15]; Jo et al., 2017[17]; Kemei and Nyerere, 2016[18]) while other studies including the present study, as well as the studies by Welch et al. (2005[35]) and Cheng et al. (2012[12]) have reported cuts and lacerations to be the most prevalent injuries. There are several reasons for the contradictory results of these studies. One reason is data collection method. In the present study, data was collected using self-reporting method while in the above-mentioned studies, data was extracted from injury database. Therefore, non-serious injuries might have not been recorded in the database. On the other hand, workers who have been seriously injured during working at height might have not been included in the population under investigation. In addition, different study results can be due to diversity and number of occupational groups surveyed in the construction industry. For example, cuts and lacerations are more prevalent among workers who directly use machinery and sharp hand tools.

Studies have shown that some characteristics such as age, level of education, marital status, work experience, training and supervision and the use of personal protective equipment affect the prevalence of WRII among construction workers, which is consistent with the results of the present study (Mersha et al., 2017[22]; Savage, 1993[28]). In the present study, the number of workers in the workplace and the employment status also affected the prevalence of WRII. The high prevalence of occupational accidents among young workers and those with little work experience is probably due to their low skill levels and lack of compliance with occupational safety and health requirements in the workplace. The highest prevalence rate of WRII was reported in small workplaces and among seasonal workers. The possible explanation for this report is that health care administration officers pay less attention to the safety and health status in these construction workplaces compared with large industries in Iran. The high prevalence rate of work-related injuries and illnesses among seasonal workers can be due to the occasional substitute and the use of less experienced young workers with lower wages as well as less responsibilities for the provision of occupational health services by employers. In this study, as in other studies (Boschman et al., 2013[9]; Holmström, 1992[16]; Siu et al., 2004[29]; Wang et al., 2017[34]), musculoskeletal disorders (53, 3 %) were the most prevalent work-related illnesses among construction workers. This finding is consistent with the fact that workers have reported ergonomic factors (85.3 %) including prolonged standing and inappropriate working postures as the most harmful factors in their workplace. Eye disease (43.1 %) was the next most prevalent work-related illness among construction workers. 

In some studies, including the study by Alazab (2004[3]) on construction workers in Egypt, the prevalence rate of eye diseases was higher than that of musculoskeletal disorders due to lack of use of personal protective equipment such as glasses. This difference may also be due to the difference in the type of construction activities and data collection methods.

Bhuiyan et al. (2016[6]) reported skin diseases (59.5 %) as the most prevalent diseases among construction workers. This is not consistent with the results of the present study and the difference in results can be due to clothing contamination or the use of personal protective equipment such as gloves. It is also noteworthy that high prevalence of fungal infections in Bangladesh due to warm and humid weather can have a significant effect on the prevalence of work-related skin diseases among construction workers who work in open spaces.

The prevalence rate of work-related mental illness in this study was 27 % which accounted for a high proportion of work-related diseases compared with other studies (Bock et al., 2003[8]; Boschman et al., 2013[9]). Considering that most workers in this study were married and seasonal workers, one can point out that work instability and great financial need play a significant role in creating these mental health problems. Based on the results, 81 % of the study population used PPE which increased their psychological stress.

The prevalence of respiratory diseases among construction workers in the present study was significantly different from that in other studies (Bock et al., 2003[8]; Sullivan et al., 1995[31]), in this study, respiratory diseases were the least common among work-related illnesses probably due to the least exposure to chemical harmful factors (31 %) in the workplace based on the workers’ self-reports. Other reasons for these differences could be attending a safety training program in addition to timely and correct use of personal protective equipment, including respiratory protectors.

There were some limitations in this study that should be taken into consideration when interpreting the results. The cross-sectional design of the study, self-reporting of collected data may not allow actual causative conclusions to be made. Furthermore, since the current research was conducted among the construction workers with conservative data, bias in the collected data may have affected the results obtained. In this study, age could be an intervening variable in proving the role of work experience in causing occupational accidents and diseases, probably due to the lack of proper selection of samples in different age groups.

## Conclusion

The results of this study showed that the prevalence rate of work-related injuries and illnesses among construction workplaces in Shiraz was 31 % and cuts and lacerations were the most frequent causes of work-related injuries. Musculoskeletal disorders (53.3 %) and eye diseases (34.1 %) were the most common work-related illnesses. Considering the high prevalence of WRII among construction workers, more stringent occupational safety and health interventions are recommended in construction workplaces.

## Conflict of interest

The author(s) declare no potential conflict of interest with respect to the research, authorship, and/or publication of this article.

## Acknowledgements

This article was supported by Shiraz University of Medical Sciences, Shiraz, Iran. 

## Figures and Tables

**Table 1:**
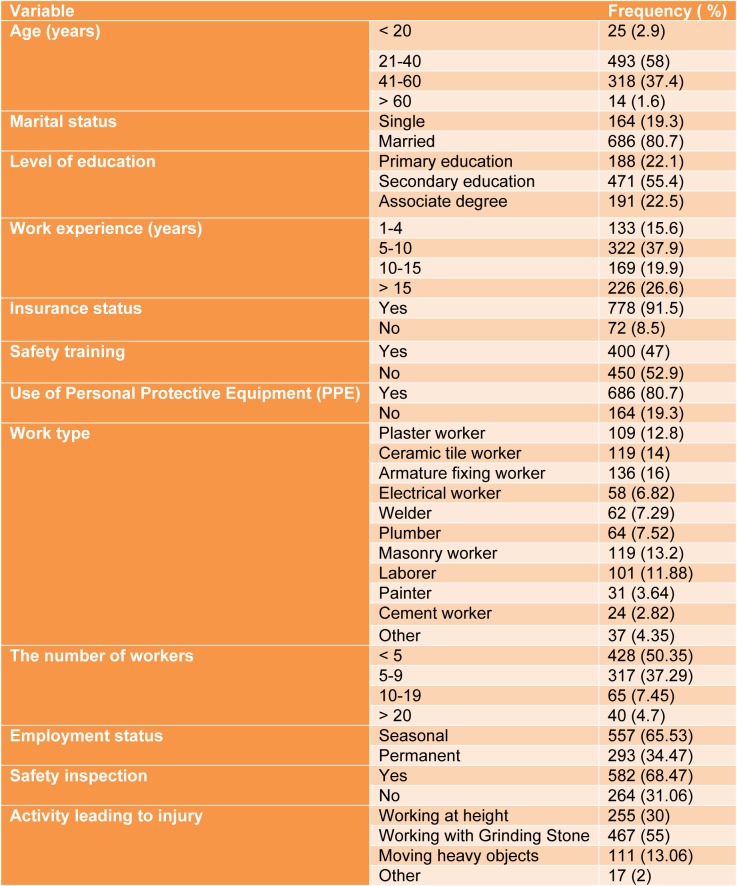
Socio-demographic characteristics of selected construction workers (N=850)

**Table 2:**
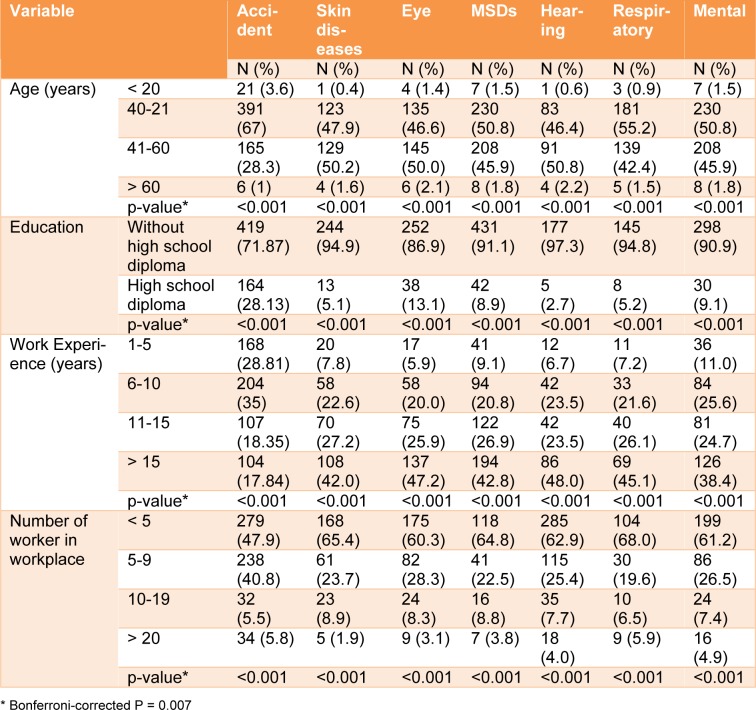
The association between overall number of accidents, work-related illnesses and demographic characteristics of the construction workers

**Table 3:**
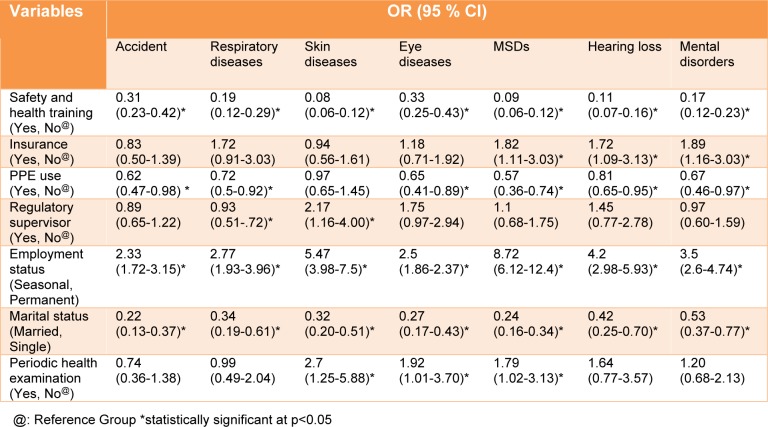
Risk ratios of exposure to WRII based on demographic characteristics
